# Relationship between adiponectin and intramuscular fat content determined by ultrasonography in older adults

**DOI:** 10.1371/journal.pone.0262271

**Published:** 2022-01-04

**Authors:** Maya Hioki, Nana Kanehira, Teruhiko Koike, Akira Saito, Kiyoshi Shimaoka, Hisataka Sakakibara, Yoshiharu Oshida, Hiroshi Akima

**Affiliations:** 1 Graduate School of Medicine, Nagoya University, Nagoya, Aichi, Japan; 2 Department of Health and Nutrition, Tokaigakuen University, Nagoya, Aichi, Japan; 3 Research Center of Health, Physical Fitness & Sports, Nagoya University, Nagoya, Aichi, Japan; 4 Center for Health and Sports Science, Kyushu Sangyo University, Fukuoka, Fukuoka, Japan; 5 Department of Human Wellness, Tokaigakuen University, Miyoshi, Aichi, Japan; University of Hawai’i at Manoa College of Tropical Agriculture and Human Resources, UNITED STATES

## Abstract

Age-associated intramuscular adipose tissue (IntraMAT) deposition induces the development of insulin resistance and metabolic syndrome. However, the relationship between IntraMAT and biochemical parameters in older adults remains unclear. The purpose of this study, therefore, was to elucidate the relationship between adiponectin and echo intensity–estimated IntraMAT using ultrasonography in normal-weight older adults (men 9, women 13) and examine biochemical parameters. Blood tests were performed to determine fasting levels of glucose, insulin, hemoglobin A1c, total cholesterol (Total-C), high-density-lipoprotein cholesterol, low-density-lipoprotein cholesterol (LDL-C), free fatty acid, triglycerides (TGs), adiponectin, leptin, high-sensitivity C-reactive protein, and high-sensitivity tumor necrosis factor, and homoeostasis model assessment index of insulin resistance (HOMA-IR). Mean gray-scale echo intensity was calculated as the IntraMAT index of the vastus lateralis. Waist circumference was measured at the level of the navel as the visceral adipose tissue (VAT) index. Echo intensity was significantly inversely correlated with adiponectin or LDL-C, and that was significantly positively correlated with TG. Adiponectin level was inversely correlated with waist circumference. Partial correlation analysis with waist circumference as the control variable revealed that adiponectin was inversely correlated with echo intensity, independent of waist circumference, whereas no such correlation was observed after controlling for LDL-C and TG levels. When biochemical parameters were grouped in the principal component analysis, among men, Total-C, insulin, and HOMA-IR or hemoglobin A1c, and high-sensitivity tumor necrosis factor–alpha were grouped with the same distribution for factors 1 and 2. Among women, glucose, insulin, HOMA-IR, and Total-C or TGs were grouped with the same distribution for factors 1 and 2. These data suggest that adiponectin level is related to IntraMAT content, independent of VAT in normal-weight older adults. The dynamics of adiponectin might not be similar to those of other circulating biochemical parameters in older men and women.

## Introduction

The lipotoxic effects of over-nutrition and corresponding increased incidence of obesity and type 2 diabetes have become a critical public health problem, particularly in developed countries [1]. Although triglycerides (TGs) are redistributed from hepatocytes and muscle cells to subcutaneous adipose tissue with advancing age, TGs can accumulate in adipose tissue in harmful ectopic locations (e.g., in the abdomen, liver, heart, and skeletal muscle). Such ectopic adipose deposits in organs can cause metabolic dysfunction [2]. Ectopic adipose tissue deposition in skeletal muscle is particularly important in humans because skeletal muscle is the largest organ. Muscular ectopic adipose tissue consists of extra-myocellular adipocytes and is designated intramuscular adipose tissue (IntraMAT), which accumulates as adipose tissue within a single muscle. Excessive IntraMAT accumulation may also lead to the development of insulin resistance and other features of metabolic syndrome [3]. Moreover, IntraMAT infiltration that occurs with aging can induce dysfunctions such as loss of muscle strength in older adults [4], thus increasing the risk of falls and fractures and the chance an individual could be become bedridden.

Ultrasound imaging is a widely available, non-invasive imaging technique frequently used for the quantitative measurement of IntraMAT. Echo intensity–estimated IntraMAT content agrees with that determined using magnetic resonance imaging (MRI) and ^1^H-magnetic resonance spectroscopy, which are considered gold-standard methods for IntraMAT quantification. However, muscle echo intensity measures not only IntraMAT but also connective tissue [5]. Many IntraMAT studies have investigated the quadriceps femoris component, particularly the vastus lateralis (VL), using muscle biopsy or imaging diagnostic methods [6]. Because the quadriceps femoris muscle is susceptible to age-related defects and the VL occupies approximately 30% of the quadriceps femoris in young adults [7], this study used ultrasound to quantify the IntraMAT of the VL.

Previous studies have demonstrated a relationship between IntraMAT and various blood biochemistry parameters in older adults. For example, our previous MRI study showed that IntraMAT in the thigh muscles, including the quadriceps femoris, hamstrings, and adductors, is correlated with levels of blood lipoproteins (TGs or low-density-lipoprotein cholesterol [LDL-C]) [8]. However, the relationship between IntraMAT in VL and adiponectin remains unclear. Adiponectin and leptin play roles in maintaining energy homeostasis and regulating glucose and lipid metabolism. Leptin is an adipose-derived adipokine hormone that regulates fat storage and plays a role in regulating food intake as a satiety signal [9]. Leptin levels are positively correlated with body composition (body mass index, weight, and waist circumference) as well as intermuscular adipose tissue (IMAT) [10]. An animal study demonstrated that leptin induces an increase in fatty acids in skeletal muscle and a decrease in TG accumulation [11]. Adiponectin has attracted considerable research attention because of its antidiabetes and anti-atherogenic effects and is expected to become a therapeutic target in the treatment of type 2 diabetes, metabolic syndrome, and cardiovascular diseases [12].

Adiponectin plays a role in decreasing the accumulation of ectopic fat such as visceral adipose tissue (VAT), intrahepatic lipids, intramyocellular lipids (IMCLs), and IMAT (which includes both IntraMAT and adipose tissue of the subfascia between muscles) [10, 13–15]. Hence, adiponectin may also be closely related to the accumulation of IntraMAT in a manner similar to that of VAT, IMCL, and IMAT; however, the relationship between adiponectin and IntraMAT remains poorly understood. Adiponectin could play a role in determining healthy longevity in older adults. For example, the genotype and allele frequencies of the rs1501299 single-nucleotide polymorphism in the adiponectin gene was found to be associated with longevity in men [16], and higher adiponectin levels were associated with lower risk of type 2 diabetes [17]. The purpose of this study was primarily to determine whether adiponectin is correlated with echo intensity–estimated IntraMAT in non-obese older men and women and secondarily to and examine biochemical parameters. We hypothesized that adiponectin levels are inversely correlated with IntraMAT.

## Material and methods

### Participants

We recruited participants in the age range of 60 to 80 years at local exercise clubs designed specifically for older adults. Exclusion criteria were explained to potential participants in the local exercise clubs. Participant eligibility was assessed using a questionnaire (n = 27), which resulted in the exclusion of a total of 5 participants for the following reasons: did not meet inclusion criteria (n = 3), or were excluded for other reasons (n = 2). No participants who were recruited for the study declined to participate. Finally, a total of 22 participants (9 men, 13 women) between the age of 62 and 77 years were decided for the study. All participants were living independently. The clinical history of each participant was assessed using a questionnaire. Participants with a history of heart disease (myocardial infarction, angina pectoris, cardiac insufficiency), cerebrovascular disease (cerebral infarction, hemorrhage), extreme hypertension (systolic blood pressure ≥180 mm Hg; diastolic blood pressure ≥110 mm Hg), neuromuscular disorders, or limb surgery were excluded. Of the three participants with type 2 diabetes, one was receiving mitiglinide calcium hydrate, one glibenclamide and mitiglinide calcium hydrate/voglibose, and one metformin. All participants provided written informed consent prior to enrollment. The study was approved by the Ethics Committee of the Graduate School of Medicine of Nagoya University, and all protocols were in accordance with the guidelines of the Declaration of Helsinki. A portion of the data was previously reported by Hioki et al. [18].

### Study protocol

The study protocol included measurement of body composition, ultrasonography, and blood tests in the morning for all participants. Participants visited the laboratory twice to ensure reproducibility of the ultrasonography measurements, with an interval of approximately 1 week between measurements. Blood tests and dietary habit assessments were performed on different dates.

### Anthropometric characteristics

According to the U.S. National Institutes of Health recommendations, waist circumference was measured at the level of the navel as the VAT index [19]. Participants were advised to take relaxed and natural breaths before the actual waist measurement to minimize the inward pull of the abdominal contents during measurement [20]. Hip circumference was taken as the greatest circumference of the pelvis.

### Biochemical parameters

Blood tests were performed to determine the fasting levels of glucose, insulin, hemoglobin A1c, total cholesterol (Total-C), high-density-lipoprotein cholesterol (HDL-C), low-density-lipoprotein cholesterol (LDL-C), free fatty acid, triglycerides (TGs), adiponectin, leptin, high-sensitivity C-reactive protein (hs-CRP), and high-sensitivity tumor necrosis factor (hs-TNFα). Homoeostasis model assessment index of insulin resistance (HOMA-IR) was calculated as fasting glucose (mg/dL) × fasting insulin (μIU/mL)/405 [21].

### Ultrasonography

Participants visited the laboratory twice for ultrasonography testing and re-testing. Ultrasonography measurements were performed by a single investigator (MH) using a LOGUQ e instrument (GE Healthcare, Boston, MA). B-mode with a multi-frequency linear transducer (8.0–12.0 MHz) was used. The transducer frequency and gain were adjusted to 8.0 MHz and 80 dB, respectively. Scanning depth was set to 8.0 cm. Participants were assessed in the prone position with the leg fully extended and relaxed. Ultrasonographic images of the VL (lateral) were obtained at the mid-thigh between the greater trochanter and lateral condyle of the femur. Five images were collected, and all images were stored in the ultrasonographic device in DICOM format for future analysis. Ultrasonographic images were allocated serial numbers to prevent individual identification before subsequent analysis.

### Intramuscular fat measurement by echo intensity

As an index that reflects IntraMAT, intramuscular fat content was measured based on ultrasonography echo intensity. A region of interest (ROI) was selected in the image of each VL, including as much of the muscle as possible, and bone and surrounding fascia were excluded. The smoothing function was applied to decrease noise in the ROI. Mean echo intensity of the ROI was calculated (8-bit resolution, resulting in a value between 0 and 255; scale: black = 0; white = 255). Mean echo intensity within the ROI in five images was measured for the VL, and five images with highest and lowest echo intensity values were excluded to minimize variations resulting from technical errors. The echo intensity of three remaining images was averaged for future analysis.

### Muscle thickness and lateral subcutaneous thickness

Muscle and subcutaneous tissue thicknesses were measured with electronic calipers placed at the middle of the ultrasound image. Muscle thickness of the VL was measured between the superficial and ventral muscle fascia, and subcutaneous tissue thickness was measured between the uppermost part of the skin and the superficial fascia of the muscle at the lateral site. Three images were scanned for each part of the thigh, and these images were averaged for future analysis.

### Physical activity levels and dietary habits

Time spent performing physical activities and total steps taken over a 10-day period were estimated from three-dimensional ambulatory accelerometer (Lifecorder; Suzuken Co., Nagoya, Japan) records, and intensity was categorized as light (<3.0 metabolic equivalents [METs]), moderate (3.0–6.0 METs), or vigorous (>6.0 METs). Physical activity level was calculated as the product of METs and time spent performing physical activities (MET h) at each intensity level (see previous studies [22, 23] for additional details). The dietary habits of the participants were assessed by a nutritionist. Dietary habits were estimated using a food frequency questionnaire [24], which included 39 food and beverage items. The questionnaire asked participants about their average intake and frequency of consumption of each food. Consumption was categorized as small, medium, or large. Five categories were used to describe consumption frequency (almost always, often, sometimes, rarely, or never).

### Statistical analyses

Differences between the male and female participants were evaluated using the unpaired Student’s *t* test. Differences in biochemical parameters (glucose, insulin, HbA1c, Total-C, HDL-C, LDL-C, FFAs, TGs, adiponectin, leptin, hs-CRP, hs–TNFα, and HOMA-IR) were then assessed using principal component (PC) analysis (PCA). To simplify interpretation of the biochemical profile data for men or women, we used PCA because it best explained the variance of the data. Data were arranged into a *P* × *N* matrix A, where *P* = 13 biochemical parameters, and *N* = 9 or 13 (i.e., number of subjects). The covariance matrix B was calculated from the data A, and the PC weightings were determined from the eigenvectors of covariance matrix B. Extracted components with eigenvalues >1 were considered significant. Similarity of biochemical parameters was evaluated for men and women by calculating the PC weighting of the first (PC1) and second (PC2) PCs. A PC weighting >0.8 was considered significant [25]. The relationships between echo intensity, adiponectin, leptin, body composition, and biochemical parameter values were examined using Pearson’s correlation analysis. When a significant correlation between adiponectin or echo intensity and body composition or biochemical parameter values was found, partial correlations adjusted for these factors were used to quantify the correlation between adiponectin and echo intensity. Ultrasound test-retest data (the first and second measurements) and the interrater reliability of echo intensity measurements were analyzed using coefficients of variation (CVs) and 1-way intra-class correlation coefficients (ICCs). All statistical analyses were performed using SPSS software, version 24.0 (SPSS Inc., Chicago, IL). Data are presented as mean ± SD. *P*<0.05 was considered significant.

## Results

The physical characteristics, blood biochemistry data, and skeletal muscle parameters of the participants are provided in Table 1. Height and weight were higher in men than women. The percentages of body fat, leptin, and lateral subcutaneous thickness were higher in women than men.

**Table 1:** Participant characteristics.

	Men	Women	*P*-value
No. of participants	9	13	
**Physical characteristics**			
Age (years)	69.4 ± 4.3	68.2 ± 4.6	0.54
Height (cm)	167.6 ± 7.2	151.5 ± 4.2	0.00
Weight (kg)	62.1 ± 7.9	51.5 ± 7.6	0.00
BMI (kg·m^−2^)	22.0 ± 1.6	22.4 ± 2.7	0.70
Waist circumference (cm)	83.9 ± 4.2	81.0 ± 9.5	0.42
Hip circumference (cm)	92.7 ± 4.0	91.4 ± 5.6	0.57
WHR	0.9 ± 0.0	0.9 ± 0.1	0.39
Fat (%)	23.7 ± 2.5	33.6 ± 4.8	0.00
**Blood biochemistry**			
Fasting glucose (mg/dL)	103.4 ± 24.2	90.4 ± 10.5	0.09
Fasting insulin (μIU/mL)	8.0 ± 7.5	5.6 ± 2.6	0.28
HbA1c (%)	5.8 ± 0.4	6.0 ± 0.4	0.42
Total-C (mg/dL)	220.1 ± 31.6	214.8 ± 29.5	0.68
HDL-C (mg/dL)	60.6 ± 15.1	60.5 ± 14.5	0.98
LDL-C (mg/dL)	133.4 ± 26.3	130.8 ± 22.5	0.80
FFAs (μEq/L)	728.7 ± 203.1	731.5 ± 223.4	0.97
TGs (mg/dL)	111.3 ± 89.4	87.1 ± 38.3	0.39
Adiponectin (μg/mL)	10.1 ± 4.7	14.0 ± 10.7	0.32
Leptin (ng/mL)	4.3 ± 1.1	8.1 ± 4.0	0.01
hs-CRP (ng/mL)	741.1 ± 772.4	4678.5 ± 13403.6	0.39
hs-TNFα (pg/mL)	1.6 ± 0.9	1.2 ± 0.3	0.20
HOMA-IR	2.1 ± 2.0	1.3 ± 0.7	0.19
**Skeletal muscle profiles**			
Echo intensity (a.u.)	70.3 ± 5.9	70.0 ± 6.7	0.92
Muscle thickness of VL (cm)	2.0 ± 0.2	1.7 ± 0.4	0.06
Lateral subcutaneous thickness (cm)	0.4 ± 0.1	0.7 ± 0.3	0.00

Value are mean ± SD. BMI, body mass index; FFAs, free fatty acids; HbA1c, hemoglobin A1c; HDL-C, high-density-lipoprotein cholesterol; HOMA-IR, homoeostasis model assessment index of insulin resistance; hs-CRP, high-sensitivity C-reactive protein; hs-TNFα, high-sensitivity tumor necrosis factor–alpha; LDL-C, low-density-lipoprotein cholesterol; TGs, triglycerides; Total-C, total cholesterol; VL, vastus lateralis; WHR, waist-to-hip ratio. Only one male subject had a hs-CRP value <50 ng/mL; therefore, the value was taken as 50 ng/mL.

**Fig 1:**
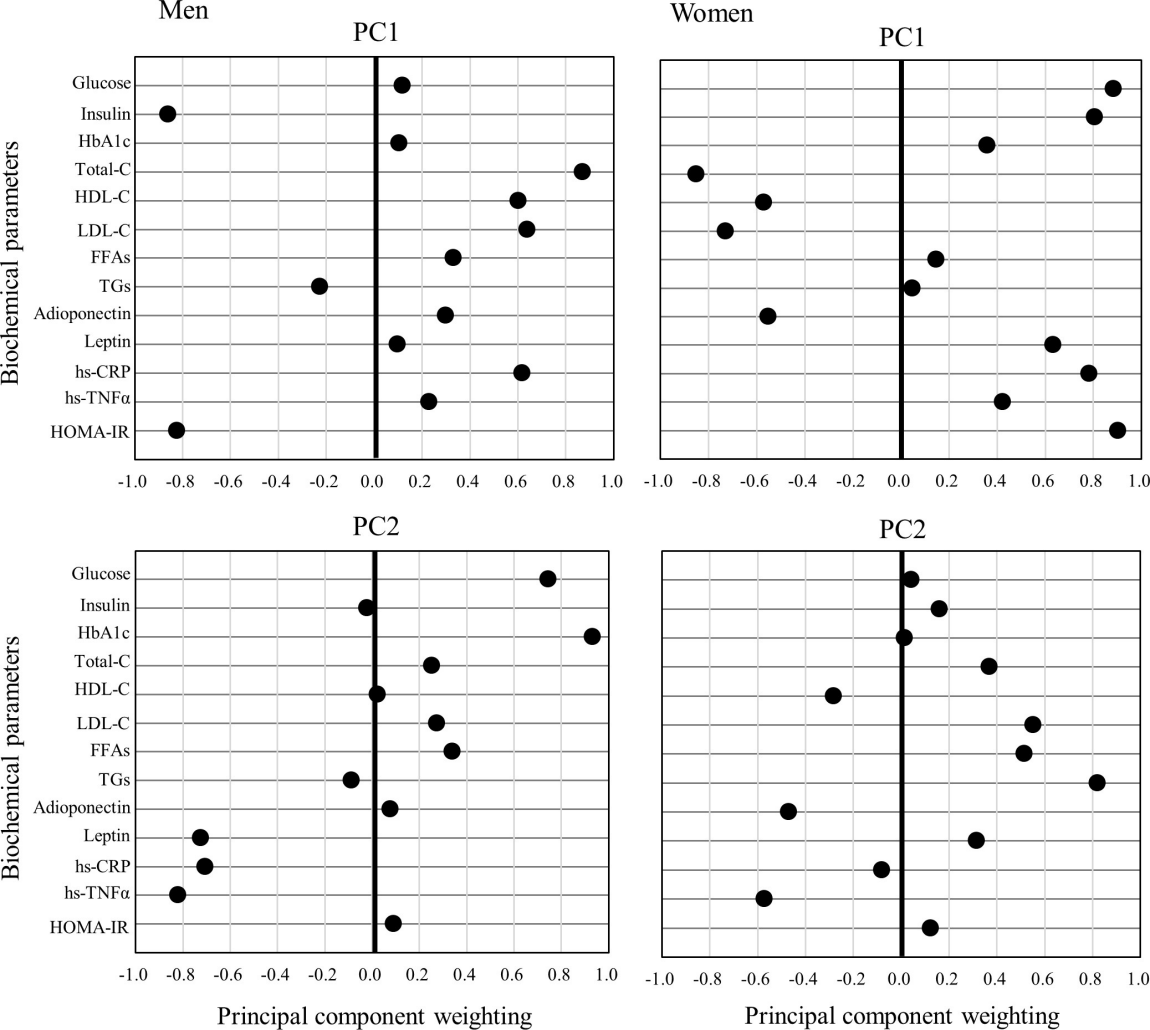
PC weighting in men (a, left side) and women (b, right side). FFAs, free fatty acids; HbA1c, hemoglobin A1c; HDL-C, high-density-lipoprotein cholesterol; HOMA-IR, homoeostasis model assessment index of insulin resistance; hs-CRP, high-sensitivity C-reactive protein; hs-TNFα, high-sensitivity tumor necrosis factor; LDL-C, low-density-lipoprotein cholesterol; TGs, triglycerides; Total-C, total cholesterol. PC weighting was significant (>0.8).

PCA identified 4 factors (84.4%) in men and 5 factors (88.5%) in women. Fig 1 shows a significant PC weighting for factor 1 in the positive Total-C or negative insulin and HOMA-IR (28.2%) and for factor 2 in the positive HbA1c or negative hs-TNFα (26.1%) among the men, and for factor 1 in positive glucose, insulin, and HOMA-IR or negative Total-C (42.2%) and for factor 2 in positive TGs among the women (16.6%).

The physical activity and dietary habit characteristics of the participants are summarized in Table 2.

**Table 2:** Physical activity and dietary habit characteristics.

	Men	Women	*P*-value
No. of participants (men/women)	9	13	
Physical activity			
Number of steps	10389.9 ± 2465.5	9663.4 ± 2684.1	0.52
Light (min)	67.1 ± 12.2	70.2 ± 17.8	0.66
Moderate (min)	35.9 ± 22.5	29.2 ± 17.0	0.43
Vigorous (min)	2.8 ± 3.1	1.5 ± 1.3	0.26
Total (min)	105.8 ± 20.2	100.8 ± 26.7	0.64
Light (MET h)	2.6 ± 0.5	2.6 ± 0.7	0.79
Moderate (MET h)	2.5 ± 1.5	2.0 ± 1.2	0.46
Vigorous (MET h)	0.3 ± 0.4	0.2 ± 0.1	0.24
Total (MET h)	5.3 ± 1.4	4.8 ± 1.5	0.41
**Habitual dietary intake**			
Energy (kcal/body weight)	33.7 ± 5.8	36.4 ± 8.6	0.41
Carbohydrates (g/body weight)	4.6 ± 0.6	4.9 ± 1.1	0.51
Protein (g/body weight)	1.2 ± 0.3	1.4 ± 0.4	0.20
Fat (g/body weight)	0.9 ± 0.3	1.2 ± 0.3	0.08

Value are mean ± SD. MET h, metabolic equivalent × hours.

The CV and ICC of the first and second echo intensity measurements were 5.3 ± 3.7% (mean ± SD) and 0.88, respectively.

Table 3 shows Pearson correlation coefficients for echo intensity, adiponectin, leptin, and body composition or biochemical parameter values. Echo intensity was correlated with Total-C, HDL-C, LDL-C, TGs, and muscle thickness but not other body composition or blood biochemistry factors. Adiponectin was correlated with waist circumference and lateral subcutaneous thickness but not other body composition or blood biochemistry factors. Leptin was correlated with body mass index (BMI), percent body fat, and hs-CRP but not other body composition or blood biochemistry factors.

**Table 3:** Pearson correlation coefficients for echo intensity, adiponectin, and leptin and body composition or biochemical parameter values.

	Echo intensity	Adiponectin	Leptin
Age	0.10	0.15	−0.07
BMI	0.01	−0.28	0.51*
Waist circumference	0.03	−0.46*	0.15
% Fat	0.15	−0.12	0.66**
Glucose	0.20	−0.34	−0.05
Insulin	0.18	−0.31	0.02
HbA1c	0.30	−0.21	0.02
Total-C	−0.43*	0.26	−0.29
HDL-C	−0.49*	0.40	−0.19
LDL-C	−0.47*	0.12	−0.15
FFAs	0.15	−0.15	0.18
TGs	0.52*	−0.32	−0.07
hs-CRP	0.17	−0.15	0.77**
HOMA-IR	0.20	−0.33	0.03
hs-TNFα	−0.07	−0.08	−0.09
Lateral subcutaneous thickness	−0.33	0.42*	0.34
Muscle thickness	−0.56**	0.24	−0.12

*, p < 0.05

**p < 0.01.

BMI, body mass index; FFAs, free fatty acids; HbA1c, hemoglobin A1c; HDL-C, high-density-lipoprotein cholesterol; HOMA-IR, homoeostasis model assessment index of insulin resistance; hs-CRP, high-sensitivity C-reactive protein; hs-TNFα, high-sensitivity tumor necrosis factor α; LDL-C, low-density-lipoprotein cholesterol; TGs, triglycerides; Total-C, total cholesterol.

**Fig 2:**
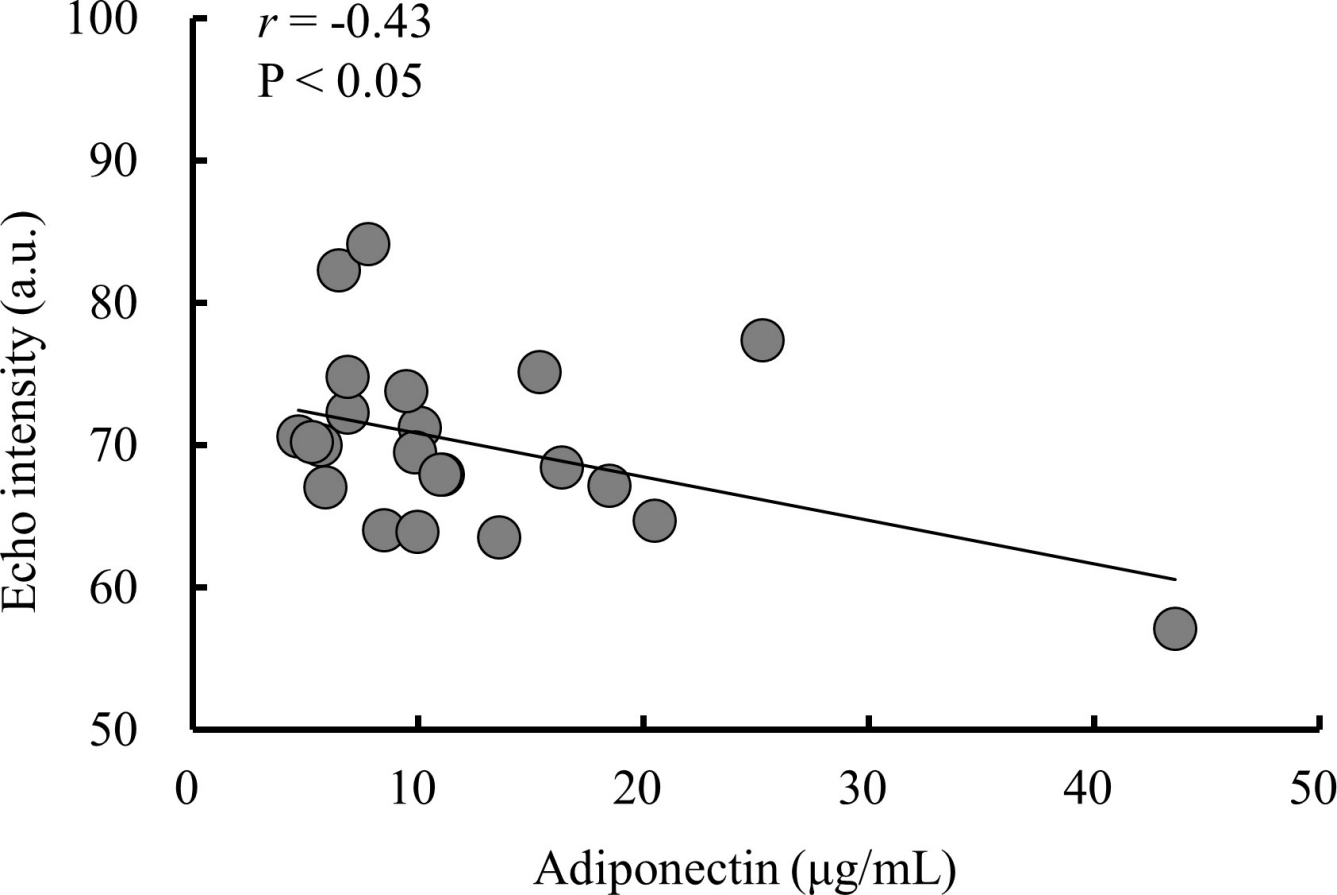
Relationship between echo intensity and adiponectin.

The relationship between echo intensity and adiponectin is shown in Fig 2. Echo intensity was significantly correlated with adiponectin but not leptin (*r* = 0.04, *P* = 0.86).

Table 4 shows partial correlation coefficients between adiponectin and echo intensity, with waist circumference, TGs, LDL-C, lateral subcutaneous thickness, and muscle thickness of VL as control variables. After controlling for waist circumference, a significant inverse association between adiponectin and echo intensity remained, whereas after controlling for TGs, LDL-C, lateral subcutaneous thickness, and muscle thickness, this correlation was not observed.

**Table 4:** Partial correlation coefficients between adiponectin and echo intensity.

Control variables	*r*	*P*-value
Waist circumference	−0.47	0.03
TGs	−0.33	0.14
LDL-C	−0.43	0.05
Lateral subcutaneous thickness	−0.34	0.12
Muscle thickness	−0.37	0.09

LDL-C, low-density-lipoprotein cholesterol; TGs, triglycerides.

## Discussion

In the present study, we demonstrated that adiponectin level was inversely correlated with echo intensity–estimated IntraMAT in the VL in normal-weight older men and women (age range 62–77 years). After controlling for waist circumference (VAT index), there remained a significant inverse association between adiponectin level and echo intensity. Furthermore, our study used PCA to examine biochemical parameters. Among men, Total-C, insulin, and HOMA-IR or HbA1c and hs-TNFα were grouped with the same distribution for factors 1 and 2. Among women, glucose, insulin, HOMA-IR, and Total-C or TGs were grouped with the same distribution for factors 1 and 2. Our results suggest that adiponectin is related to IntraMAT content, independent of VAT, and that the dynamics of adiponectin might not be similar to those of other biochemical parameters in older men and women.

Our analyses revealed an inverse correlation between adiponectin and echo intensity, independent of waist circumference. This result is comparable with reports indicating that adiponectin is inversely correlated with IMCL, independent of VAT and total body fat mass in obese women [13]. Adiponectin receptor 1 (AdipoR1) and adiponectin receptor 2 (AdipoR2) mediate increased AMP-activated kinase and PPAR activity, thereby regulating glucose and lipid metabolism [12]. In an animal study, peripheral administration of adiponectin attenuated body weight gain and reduced VAT [26]. Moreover, a single injection of purified recombinant human adiponectin in diabetic swine produced a 2- to 3-fold elevation in the circulating adiponectin level, in turn triggering a transient decrease in basal glucose level, independent of insulin level [27]. These results suggest that adiponectin mediates accumulation of VAT. According to the Framingham cohort study, adiponectin level is inversely related to ectopic fat deposition (visceral, epicardial, and mediastinal fat) [28]. Our findings thus suggest that adiponectin level is related to IntraMAT content in normal-weight older men and women, with adiponectin exhibiting a similar influence on ectopic fat (i.e., IMCL, VAT, IMAT, epicardial and mediastinal fat) [10, 13, 29]. By contrast, according to the Cardiovascular Health Study, an epidemiologic investigation (participants >80 years of age; N = 988), adiponectin levels increase with age, which is associated with increased physical disability and mortality. This phenomenon may be associated with age-related homeostatic dysregulation [30]. Adiponectin resistance can occur in persons ≥80 years old. The age of participants differed between the present study (range 62 to 77 years) and the previous study (>80 years). Therefore, our result might not agree with those of Kizer et al. [30].

The level of methylation of adiponectin-gene DNA in subcutaneous adipose tissue and VAT was found to be positively associated with LDL-C level, suggesting a common epigenetic regulation mechanism that is independent of the biological roles of these factors [31]. Moreover, adiponectin might serve as a starvation signal released by adipocytes, thereby inducing local expansion of TG stores in adipose tissue [32] and effectively redistributing TGs from hepatocytes and muscle cells to subcutaneous adipose tissue. The results of these two previous studies indicate that adiponectin controls lipogenesis and lipolysis of TGs and LDL-C. We found that adiponectin was inversely correlated with echo intensity–estimated IntraMAT, but this correlation became insignificant after adjusting for TG and LDL-C levels. These results suggest that TGs and LDL-C affect the relationship between adiponectin and IntraMAT content. When biochemical parameters were grouped in the PC analysis, among men, Total-C, insulin, and HOMA-IR or HbA1c and hs-TNFα were grouped with the same distribution for factors 1 and 2. Among women, glucose, insulin, HOMA-IR, and Total-C or TG were grouped with the same distribution for factors 1 and 2. These results indicate that factors differ between men and women. As adiponectin did not belong to the same group of factors, it might function independently.

Adiponectin is expressed more in VAT than subcutaneous adipose tissue, and VAT adipocytes are more metabolically active and more sensitive to lipolysis than subcutaneous adipose tissue adipocytes [33]. An epidemiologic investigation of Japanese middle-aged men reported that adiponectin levels were inversely correlated with VAT and subcutaneous adipose tissue in a regression model that concomitantly included these factors. VAT exhibited a significant inverse and subcutaneous adipose tissue a significant positive association [34]. These results indicate that VAT and subcutaneous adipose tissue are differentially correlated with adiponectin concentration. We also found that adiponectin level was positively correlated with subcutaneous adipose tissue; however, adiponectin level was inversely correlated with echo intensity–estimated IntraMAT. Therefore, our results are congruent with those reported by Nakamura et al. [34].

In general, older adults reportedly have lower circulating levels of leptin compared with young adults. Obesity has also been associated with leptin resistance, which can result in increased circulating leptin levels but decreased leptin signaling [35]. Moreover, plasma leptin is correlated with body fat content in young men and women, but no such correlation has been observed in middle-aged and older men and women [36]. According to Vella et al. [37], physical activity may positively affect levels of select adiposity-associated inflammatory markers (leptin, IL-6, and resistin), irrespective of total and/or central adiposity. The level of physical activity (number of steps) among older participants in the present study (men, mean 10389.9 steps; women, mean 9663.4 steps) was similar to that of young adults in our previous study (young men and women, mean 9097.4 steps). These results indicate that the older adults in our present study were quite physically active. In line with previous observations in young adult men and women [36], our present study identified a correlation between leptin and BMI or percent body fat in the older men and women enrolled in the study.

Our study has some limitations. First, VAT was estimated from waist circumference as the VAT index. Notably, we estimated this value and did not measure it. Waist circumference tended to correlate more significantly with MRI-measured VAT (n = 1192; *r* = 0.80) [19]. Waist circumference is utilized as an index of central obesity, as recommended by the U.S. National Institutes of Health, World Health Organization [20], American Heart Association, and International Diabetes Foundation. The waist-to-hip ratio and waist measurements can be utilized as a “central obesity or visceral fat index” for screening [38]. Second, our study included both men and women as a single group. Circulating levels of adiponectin and leptin are known to exhibit sexual dimorphism [12, 39]. Levels of circulating adiponectin and leptin tend to be higher in women than men, suggesting that sex hormones regulate the production of adiponectin and leptin. The PCA clearly indicated that Total-C, insulin, and HOMA-IR or HbA1c and hs-TNFα were grouped with the same distribution for factors 1 and 2 among the men, and glucose, insulin, HOMA-IR, and Total-C or TGs were grouped with the same distribution for factors 1 and 2 among the women. Biochemical parameters also likely differ between adult men and women. Our results also indicated that adiponectin and leptin levels were higher in women than men, although the difference in adiponectin levels was not significant. Third, the transmission of ultrasound beams through tissue can be attenuated due to reflection, dispersion, or absorption of the sound waves, thus reducing the echo intensity of deeper structures [40]. The non-invasive tauonic ultrasonography technique is widely used to evaluate muscle quality and quantity (i.e., muscle mass or echo intensity–estimated IntraMAT content) in both research and medical settings. However, the range of ultrasonographic images covers only a part of the muscle; therefore, IntraMAT content must be estimated from a narrow range of images.

## Conclusion

In conclusion, we assessed biochemical parameters and echo intensity–estimated IntraMAT in the VL via ultrasonography among normal-weight older adult men and women. Partial correlation analysis with waist circumference as a control variable revealed that adiponectin is inversely correlated with echo intensity, independent of waist circumference (measured as the VAT index); however, after controlling for LDL-C and TGs, this correlation was not observed. Our results thus suggest that adiponectin is related to IntraMAT content, independent of VAT. Furthermore, our study used PCA to examine biochemical parameters. Among men, Total-C, insulin, and HOMA-IR or HbA1c and hs-TNFα were grouped with the same distribution for factors 1 and 2. Among women, glucose, insulin, HOMA-IR, and Total-C or TGs were grouped with the same distribution for factors 1 and 2. The dynamics of adiponectin might not be similar to those of other circulating biochemical parameters in older men and women. The prevalence of both type 2 diabetes and obesity increases with age, and therefore, the risk of metabolic diseases increases with fat accumulation in ectopic fat deposits (i.e., IMCL, IMAT, visceral, epicardial, and mediastinal fat). IntraMAT might also be an adiponectin target tissue, suggesting that adiponectin positively affects insulin sensitivity in older adults by decreasing IntraMAT content. In addition, adiponectin might function differently in men and women.

## Supporting information

S1 Fig PC weighting among men (left side; PC1-PC4) and women (right side; PC1-PC5). FFAs, free fatty acids; HbA1c, hemoglobin A1c; HDL-C, high-density-lipoprotein cholesterol; HOMA-IR, homoeostasis model assessment index of insulin resistance; hs-CRP, high-sensitivity C-reactive protein; hs-TNFα, high-sensitivity tumor necrosis factor–alpha; LDL-C, low-density-lipoprotein cholesterol; TGs, triglycerides; Total-C, total cholesterol. PC weighting is significant (>0.8).
(TIF)

## References

[1] Stern JH, Rutkowski JM, Scherer PE. Adiponectin, Leptin, and Fatty Acids in the Maintenance of Metabolic Homeostasis through Adipose Tissue Crosstalk. Cell Metab. 2016;23(5):770–84. Epub 2016/05/12. doi: 10.1016/j.cmet.2016.04.011 ; PubMed Central PMCID: PMC4864949. 27166942 PMC4864949

[2] Szendroedi J, Roden M. Ectopic lipids and organ function. Curr Opin Lipidol. 2009;20(1):50–6. Epub 2009/01/13. doi: 10.1097/mol.0b013e328321b3a8 . 19133412

[3] Tchkonia T, Thomou T, Zhu Y, Karagiannides I, Pothoulakis C, Jensen MD, et al. Mechanisms and metabolic implications of regional differences among fat depots. Cell Metab. 2013;17(5):644–56. Epub 2013/04/16. doi: 10.1016/j.cmet.2013.03.008 ; PubMed Central PMCID: PMC3942783. 23583168 PMC3942783

[4] Visser M, Goodpaster BH, Kritchevsky SB, Newman AB, Nevitt M, Rubin SM, et al. Muscke mass, muscle strength, and muscle fat infiltration as predictors of incident mobility limitations on well-functioning or older persons. J Gerontol A Biol Sci Med Sci. 2005;60:324–33. doi: 10.1093/gerona/60.3.324 15860469

[5] Pillen S, Tak RO, Zwarts MJ, Lammens MM, Verrijp KN, Arts IM, et al. Skeletal muscle ultrasound: correlation between fibrous tissue and echo intensity. Ultrasound Med Biol. 2009;35(3):443–6. Epub 2008/12/17. doi: 10.1016/j.ultrasmedbio.2008.09.016 . 19081667

[6] Akima H, Hioki M, Yoshiko A, Koike T, Sakakibara H, Takahashi H, et al. Intramuscular adipose tissue determined by T1-weighted MRI at 3T primarily reflects extramyocellular lipids. Magn Reson Imaging. 2016;34(4):397–403. Epub 2016/01/09. doi: 10.1016/j.mri.2015.12.038 . 26743430

[7] Akima H, Ushiyama J, Kubo J, Fukuoka H, Kanehisa H, Fukunaga T. Effect of unloading on muscle volume with and without resistance training. Acta Astronautica. 2007;60:728–36.

[8] Akima H, Yoshiko A, Hioki M, Kanehira N, Shimaoka K, Koike T, et al. Skeletal muscle size is a major predictor of intramuscular fat content regardless of age. Eur J Appl Physiol. 2015;115(8):1627–35. Epub 2015/03/12. doi: 10.1007/s00421-015-3148-2 . 25757882

[9] Fasshauer M, Bluher M. Adipokines in health and disease. Trends Pharmacol Sci. 2015;36(7):461–70. Epub 2015/05/30. doi: 10.1016/j.tips.2015.04.014 . 26022934

[10] Zoico E, Rossi A, Di Francesco V, Sepe A, Olioso D, Pizzini F, et al. Adipose tissue infiltration in skeletal muscle of healthy elderly men: relationships with body composition, insulin resistance, and inflammation at the systemic and tissue level. J Gerontol A Biol Sci Med Sci. 2010;65(3):295–9. Epub 2009/10/30. doi: 10.1093/gerona/glp155 . 19864639 PMC4051307

[11] Minokoshi Y, Kim YB, Peroni OD, Fryer LG, Müller C, Carling D, et al. Leptin stimulates fatty-acid oxidation by activating AMP-activated protein kinase. Nature. 2002;415(6869):339–43. Epub 2002/01/18. doi: 10.1038/415339a . 11797013

[12] Yamauchi T, Kadowaki T. Adiponectin receptor as a key player in healthy longevity and obesity-related diseases. Cell Metab. 2013;17(2):185–96. Epub 2013/01/29. doi: 10.1016/j.cmet.2013.01.001 . 23352188

[13] Bredella MA, Torriani M, Ghomi RH, Thomas BJ, Brick DJ, Gerweck AV, et al. Adiponectin is inversely associated with intramyocellular and intrahepatic lipids in obese premenopausal women. Obesity (Silver Spring). 2011;19(5):911–6. Epub 2010/12/15. doi: 10.1038/oby.2010.296 ; PubMed Central PMCID: PMC3607306. 21151017 PMC3607306

[14] Perseghin G, Scifo P, Danna M, Battezzati A, Benedini S, Meneghini E, et al. Normal insulin sensitivity and IMCL content in overweight humans are associated with higher fasting lipid oxidation. Am J Physiol Endocrinol Metab. 2002;283(3):E556–64. Epub 2002/08/10. doi: 10.1152/ajpendo.00127.2002 . 12169449

[15] Perseghin G, Lattuada G, De Cobelli F, Esposito A, Belloni E, Canu T, et al. Serum retinol-binding protein-4, leptin, and adiponectin concentrations are related to ectopic fat accumulation. J Clin Endocrinol Metab. 2007;92(12):4883–8. Epub 2007/11/08. doi: 10.1210/jc.2007-0325 . 17986645

[16] Khabour OF, Mesmar FS, Alatoum MA, Gharaibeh MY, Alzoubi KH. Associations of polymorphisms in adiponectin and leptin genes with men’s longevity. The aging male: the official journal of the International Society for the Study of the Aging Male. 2010;13(3):188–93. Epub 2010/03/06. doi: 10.3109/13685531003657800 . 20201642

[17] Kanaya AM, Harris T, Goodpaster BH, Tylavsky F, Cummings SR. Adipocytokines attenuate the association between visceral adiposity and diabetes in older adults. Diabetes Care. 2004;27(6):1375–80. Epub 2004/05/27. doi: 10.2337/diacare.27.6.1375 . 15161791

[18] Hioki M, Kanehira N, Koike T, Saito A, Takahashi H, Shimaoka K, et al. Effect of electromyostimulation on intramyocellular lipids of the vastus lateralis in older adults: a randomized controlled trial. BMC Musculoskelet Disord. 2021;22(1):569. Epub 2021/06/24. doi: 10.1186/s12891-021-04456-6 . 34158031 PMC8218407

[19] Fang H, Berg E, Cheng X, Shen W. How to best assess abdominal obesity. Current opinion in clinical nutrition and metabolic care. 2018;21(5):360–5. Epub 2018/06/20. doi: 10.1097/MCO.0000000000000485 ; PubMed Central PMCID: PMC6299450. 29916924 PMC6299450

[20] WHO. Waist Circumference and Waist-Hip Ratio Report of a WHO Expert Consultation. Geneva, Switzerland; World Health Organisation. 2008.

[21] Matthews DR, Hosker JP, Rudenski AS, Naylor BA, Treacher DF, Turner RC. Homeostasis model assessment: insulin resistance and beta-cell function from fasting plasma glucose and insulin concentrations in man. Diabetologia. 1985;28(7):412–9. Epub 1985/07/01. doi: 10.1007/BF00280883 . 3899825

[22] Kumahara H, Schutz Y, Ayabe M, Yoshioka M, Yoshitake Y, Shindo M, et al. The use of uniaxial accelerometry for the assessment of physical-activity-related energy expenditure: a validation study against whole-body indirect calorimetry. Br J Nutr. 2004;91(2):235–43. Epub 2004/02/06. doi: 10.1079/BJN20031033 . 14756909

[23] Hioki M, Kanehira N, Koike T, Saito A, Takahashi H, Shimaoka K, et al. Relationship between physical activity and intramyocellular lipid content is different between young and older adults. Eur J Appl Physiol. 2019;119(1):113–22. Epub 2018/10/12. doi: 10.1007/s00421-018-4005-x . 30306258

[24] Perez Rodrigo C, Aranceta J, Salvador G, Varela-Moreiras G. Food frequency questionnaires. Nutr Hosp. 2015;31 Suppl 3:49–56. Epub 2015/02/27. doi: 10.3305/nh.2015.31.sup3.8751 . 25719771

[25] Cohen AA, Milot E, Li Q, Bergeron P, Poirier R, Dusseault-Bélanger F, et al. Detection of a novel, integrative aging process suggests complex physiological integration. PLoS One. 2015;10(3):e0116489. Epub 2015/03/12. doi: 10.1371/journal.pone.0116489 ; PubMed Central PMCID: PMC4356614 general professional competing interest of being required to publish research articles for career advancement and obtaining funding. 25761112 PMC4356614

[26] Masaki T, Chiba S, Yasuda T, Tsubone T, Kakuma T, Shimomura I, et al. Peripheral, but not central, administration of adiponectin reduces visceral adiposity and upregulates the expression of uncoupling protein in agouti yellow (Ay/a) obese mice. Diabetes. 2003;52(9):2266–73. Epub 2003/08/28. doi: 10.2337/diabetes.52.9.2266 . 12941765

[27] Hu X, She M, Hou H, Li Q, Shen Q, Luo Y, et al. Adiponectin decreases plasma glucose and improves insulin sensitivity in diabetic Swine. Acta biochimica et biophysica Sinica. 2007;39(2):131–6. Epub 2007/02/06. doi: 10.1111/j.1745-7270.2007.00255.x . 17277888

[28] Jain SH, Massaro JM, Hoffmann U, Rosito GA, Vasan RS, Raji A, et al. Cross-sectional associations between abdominal and thoracic adipose tissue compartments and adiponectin and resistin in the Framingham Heart Study. Diabetes Care. 2009;32(5):903–8. Epub 2009/02/19. doi: 10.2337/dc08-1733 ; PubMed Central PMCID: PMC2671095. 19223612 PMC2671095

[29] Kantartzis K, Rittig K, Balletshofer B, Machann J, Schick F, Porubska K, et al. The relationships of plasma adiponectin with a favorable lipid profile, decreased inflammation, and less ectopic fat accumulation depend on adiposity. Clin Chem. 2006;52(10):1934–42. Epub 2006/08/19. doi: 10.1373/clinchem.2006.067397 . 16916991

[30] Kizer JR, Arnold AM, Strotmeyer ES, Ives DG, Cushman M, Ding J, et al. Change in circulating adiponectin in advanced old age: determinants and impact on physical function and mortality. The Cardiovascular Health Study All Stars Study. J Gerontol A Biol Sci Med Sci. 2010;65(11):1208–14. Epub 2010/07/10. doi: 10.1093/gerona/glq122 ; PubMed Central PMCID: PMC2954239. 20616148 PMC2954239

[31] Houde AA, Légaré C, Biron S, Lescelleur O, Biertho L, Marceau S, et al. Leptin and adiponectin DNA methylation levels in adipose tissues and blood cells are associated with BMI, waist girth and LDL-cholesterol levels in severely obese men and women. BMC medical genetics. 2015;16:29. Epub 2015/05/02. doi: 10.1186/s12881-015-0174-1 ; PubMed Central PMCID: PMC4631085. 25929254 PMC4631085

[32] Kim JY, van de Wall E, Laplante M, Azzara A, Trujillo ME, Hofmann SM, et al. Obesity-associated improvements in metabolic profile through expansion of adipose tissue. J Clin Invest. 2007;117(9):2621–37. Epub 2007/08/25. doi: 10.1172/JCI31021 ; PubMed Central PMCID: PMC1950456. 17717599 PMC1950456

[33] Ibrahim MM. Subcutaneous and visceral adipose tissue: structural and functional differences. Obesity reviews: an official journal of the International Association for the Study of Obesity. 2010;11(1):11–8. Epub 2009/08/07. doi: 10.1111/j.1467-789X.2009.00623.x . 19656312

[34] Nakamura Y, Sekikawa A, Kadowaki T, Kadota A, Kadowaki S, Maegawa H, et al. Visceral and subcutaneous adiposity and adiponectin in middle-aged Japanese men: the ERA JUMP study. Obesity (Silver Spring). 2009;17(6):1269–73. Epub 2009/07/09. doi: 10.1038/oby.2009.3 ; PubMed Central PMCID: PMC2849631. 19584883 PMC2849631

[35] Morris DL, Rui L. Recent advances in understanding leptin signaling and leptin resistance. Am J Physiol Endocrinol Metab. 2009;297(6):E1247–59. Epub 2009/09/03. doi: 10.1152/ajpendo.00274.2009 ; PubMed Central PMCID: PMC2793049. 19724019 PMC2793049

[36] Moller N, O’Brien P, Nair KS. Disruption of the relationship between fat content and leptin levels with aging in humans. J Clin Endocrinol Metab. 1998;83(3):931–4. Epub 1998/03/20. doi: 10.1210/jcem.83.3.4620 . 9506751

[37] Vella CA, Allison MA, Cushman M, Jenny NS, Miles MP, Larsen B, et al. Physical Activity and Adiposity-related Inflammation: The MESA. Med Sci Sports Exerc. 2017;49(5):915–21. Epub 2016/12/16. doi: 10.1249/MSS.0000000000001179 ; PubMed Central PMCID: PMC5392139. 27977529 PMC5392139

[38] Gadekar T, Dudeja P, Basu I, Vashisht S, Mukherji S. Correlation of visceral body fat with waist-hip ratio, waist circumference and body mass index in healthy adults: A cross sectional study. Medical journal, Armed Forces India. 2020;76(1):41–6. Epub 2020/02/06. doi: 10.1016/j.mjafi.2017.12.001 ; PubMed Central PMCID: PMC6994756. 32020967 PMC6994756

[39] Dornbush S, Aeddula NR. Physiology, Leptin. StatPearls. Treasure Island (FL): StatPearls Publishing Copyright © 2020, StatPearls Publishing LLC.; 2020. 30725723

[40] Pillen S, Arts IMP, Zwarts MJ. Muscle ultrasound in neuromuscular disorders. Muscle Nerve. 2008;37:679–93. 18506712 10.1002/mus.21015

