# Supplementary figures and images for: Relationship between adiponectin and intramuscular fat content determined by ultrasonography in older adults
Source: PLoS One. 2022 Jan 4;17(1):e0262271. doi: 10.1371/journal.pone.0262271 (PMC8726469; doi:10.1371/journal.pone.0262271)

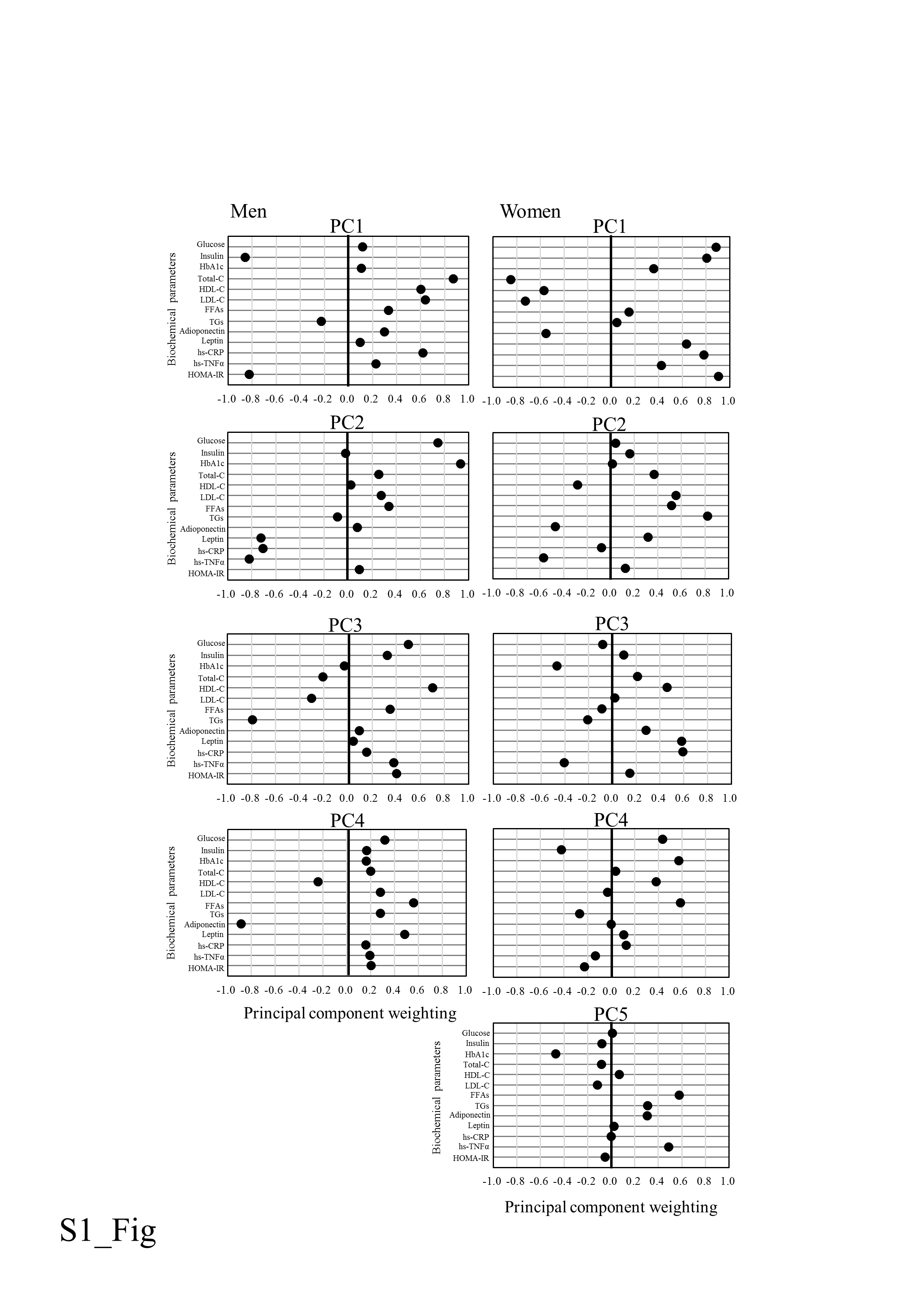

Supplement: S1 Fig — PC weighting among men (left side; PC1-PC4) and women (right side; PC1-PC5). FFAs, free fatty acids; HbA1c, hemoglobin A1c; HDL-C, high-density-lipoprotein cholesterol; HOMA-IR, homoeostasis model assessment index of insulin resistance; hs-CRP, high-sensitivity C-reactive protein; hs-TNFα, high-sensitivity tumor necrosis factor–alpha; LDL-C, low-density-lipoprotein cholesterol; TGs, triglycerides; Total-C, total cholesterol. PC weighting is significant (>0.8). (TIF) [file pone.0262271.s001.tif]
